# RLK-YOLOv8: multi-stage detection of strawberry fruits throughout the full growth cycle in greenhouses based on large kernel convolutions and improved YOLOv8

**DOI:** 10.3389/fpls.2025.1552553

**Published:** 2025-03-25

**Authors:** Lei He, Dasheng Wu, Xinyu Zheng, Fengya Xu, Shangqin Lin, Siyang Wang, Fuchuan Ni, Fang Zheng

**Affiliations:** ^1^ College of Mathematics and Computer Science, Zhejiang A&F University, Hangzhou, China; ^2^ Key Laboratory of State Forestry and Grassland Administration on Forestry Sensing Technology and Intelligent Equipment, Hangzhou, China; ^3^ Key Laboratory of Forestry Intelligent Monitoring and Information Technology of Zhejiang Province, Hangzhou, China; ^4^ Engineering Research Center of Intelligent Technology for Agriculture, Ministry of Education, Wuhan, China

**Keywords:** YOLOv8, RepLKNet, RepNCSPELAN4, DynamicHead, PolyLoss, full growth cycle of strawberry fruits

## Abstract

**Introduction:**

In the context of intelligent strawberry cultivation, achieving multi-stage detection and yield estimation for strawberry fruits throughout their full growth cycle is essential for advancing intelligent management of greenhouse strawberries. Addressing the high rates of missed and false detections in existing object detection algorithms under complex backgrounds and dense multi-target scenarios, this paper proposes an improved multi-stage detection algorithm RLK-YOLOv8 for greenhouse strawberries. The proposed algorithm, an enhancement of YOLOv8, leverages the benefits of large kernel convolutions alongside a multi-stage detection approach.

**Method:**

RLK-YOLOv8 incorporates several improvements based on the original YOLOv8 model. Firstly, it utilizes the large kernel convolution network RepLKNet as the backbone to enhance the extraction of features from targets and complex backgrounds. Secondly, RepNCSPELAN4 is introduced as the neck network to achieve bidirectional multi-scale feature fusion, thereby improving detection capability in dense target scenarios. DynamicHead is also employed to dynamically adjust the weight distribution in target detection, further enhancing the model’s accuracy in recognizing strawberries at different growth stages. Finally, PolyLoss is adopted as the loss function, which effectively improve the localization accuracy of bounding boxes and accelerating model convergence.

**Results:**

The experimental results indicate that RLK-YOLOv8 achieved a mAP of 95.4% in the strawberry full growth cycle detection task, with a precision and F1-score of 95.4% and 0.903, respectively. Compared to the baseline YOLOv8, the proposed algorithm demonstrates a 3.3% improvement in detection accuracy under complex backgrounds and dense multi-target scenarios.

**Discussion:**

The RLK-YOLOv8 exhibits outstanding performance in strawberry multi-stage detection and yield estimation tasks, validating the effectiveness of integrating large kernel convolutions and multi-scale feature fusion strategies. The proposed algorithm has demonstrated significant improvements in detection performance across various environments and scenarios.

## 1 Introduction

In recent years, the increasing demand for agricultural modernization and intelligence has made the precise detection of strawberries throughout their full growth cycle crucial. It holds the potential to significantly enhance yield estimation accuracy and optimize resource allocation (Zheng et al., 2021; Hopf et al., 2022). Strawberries, as one of the most consumed fruits globally, play a prominent role in China’s agricultural economy, with the country leading in global strawberry production (Sobekova et al., 2013; Hernández-Martínez et al., 2023). Accurate yield predictions not only assist farmers in formulating optimal planting and management strategies but also provide agricultural managers with the scientific basis for market supply, thereby contributing to sustainable agricultural development (Nassar et al., 2020). Greenhouse strawberries, as high-value products, are priced individually in the premium fruit market. Multi-stage detection of strawberries throughout their growth cycle allows for accurate predictions of the number of fruits that are likely to reach the market, which is critical for maximizing economic benefits and resource utilization efficiency (Kempler, 2002).

The main challenge in strawberry yield estimation lies in the complexity of fruit detection (Wang et al., 2022). Strawberries are typically small, densely packed, and often obscured by leaves, making detection difficult. In addition, variations in lighting, shadows, and background clutter within greenhouse environments further complicate the detection process (Zheng et al., 2021). Traditional image processing methods based on color, shape, and texture features have shown limited effectiveness, particularly in handling partial occlusion and overlapping fruit. Moreover, many traditional models rely heavily on single-task detection, which fails to account for the dynamic nature of fruit growth stages. Various camera sensors, such as RGB, RGB-depth (RGB-D), infrared (IR), and multispectral imaging, have been applied to strawberry production (Liu et al., 2014; Xiong et al., 2019; Anraeni et al., 2021). For example, Rajendra et al. (2009) developed an RGB-to-hue, saturation, and intensity (HSI) color mapping algorithm for mature strawberries, achieving 76% accuracy for unobstructed ripe strawberries, but failing to detect unripe ones. To address these limitations, multi-scale feature fusion, dynamic attention mechanisms, and large receptive fields are crucial for accurately detecting strawberries throughout their growth cycle in real-time, particularly in environments where fruits are closely clustered and partially hidden. This paper proposes RLK-YOLOv8 to improve the accuracy of multi-stage strawberry detection in complex greenhouse environments. The contributions of this study are as follows:

1. The RepLKNet module is integrated into the backbone to expand the receptive field, enhancing feature extraction and improving detection accuracy in occluded and densely clustered strawberry environments.
2. The RepNCSPELAN4 module is employed as the neck component, optimizing multi-scale feature fusion to improve the model’s ability to distinguish strawberries from complex backgrounds.
3. The DynamicHead module is introduced to enable adaptive detection across different growth stages, enhancing robustness against variations in fruit size, occlusion, and lighting conditions.
4. PolyLoss is utilized as the loss function to optimize convergence and balance detection precision across multiple strawberry growth stages, reducing false positives in cluttered greenhouse settings.

The rest of this study is organized as follows: Section 2 reviews relevant methods in strawberry detection, including recent YOLO advancements in agriculture. Section 3 describes the experimental methods and principles, focusing on YOLOv8 architecture and the proposed RLK-YOLOv8 model. Section 4 presents and analyzes the experimental results, comparing RLK-YOLOv8 with other detection algorithms. Section 5 discusses the challenges encountered throughout the study, including limitations of the dataset and the model’s performance under complex environmental conditions. Finally, Section 6 concludes the study and outlines future research directions in strawberry detection and agricultural applications.

## 2 Related work

YOLO-based models have shown impressive results in object detection tasks, but challenges remain, especially with occlusion and dense object clustering. To address these challenges, methods such as transfer learning and multi-domain adaptation have been proposed to improve feature extraction. For instance, adversarial transfer learning has been used for image segmentation across different domains, increasing model robustness in complex environments (Mishra et al., 2023). Deep learning models in crop detection are gradually replacing traditional feature extraction-based methods. Common models include R-CNN (He et al., 2017), SSD (Liu et al., 2016), RetinaNet (Lin, 2017), and the YOLO (Bochkovskiy et al., 2020) series. These models have been successfully applied to agriculture, with Transformer-based models recently improving fruit detection in complex environments. Wei et al. (2025) proposed a green apple detection method using an enhanced DETR network with multidimensional feature extraction and Transformer modules, improving detection accuracy for near-color fruits under challenging conditions. Chen et al. (2019) used drones to capture top-view images of strawberries, employing a deep neural network to estimate yield with 84.1% accuracy. Similarly, Zhang et al. (2020) used Faster R-CNN to analyze strawberry plant vigor through remote sensing images. Zhao et al. (2023) introduced an improved CR-YOLOv5s algorithm, using coordinate attention mechanisms to detect chrysanthemum buds and flowers in complex backgrounds, achieving an average accuracy of 93.9%, which forms a reliable basis for flower yield estimation. While many studies have focused on agricultural robots for large-scale farming, fewer have addressed small-scale agriculture. Fujinaga (2024) developed a multi-functional agricultural robot capable of both fruit harvesting and truss pruning, detecting cutting points through deep learning-based semantic segmentation and plant features. This highlights the importance of multi-tasking in small-scale agriculture for enhancing robot efficiency.

In strawberry detection, research has primarily focused on mature fruit for harvesting, with limited studies on detecting fruits in the flowering and early development stages. To address this, recent progress in large-kernel convolutional networks has emerged due to their ability to expand receptive fields and enhance global feature extraction. Ding et al. (2022) proposed the RepLKNet (Reparameterized Large-Kernel Network), which uses ultra-large kernels to expand the receptive field, improving efficiency by reparameterizing smaller kernels. Pei et al. (2024) employed RepLKNet in plant disease recognition, addressing the limitations of small-kernel networks and improving performance, achieving an overall accuracy of 93.6%.

This paper proposes an improved method for multi-stage detection of strawberries throughout their growth cycle, based on the You Only Look Once version 8 (YOLOv8) (Sohan et al., 2024) model, named RLK-YOLOv8 (Reparameterized Large-Kernel YOLOv8). The RLK-YOLOv8 model leverages deep CNNs to handle strawberry detection in challenging greenhouse environments. Adaptive CNNs have been shown to improve performance by capturing complex features and compensating for environmental variables such as lighting and occlusion (Babu et al., 2023). The model incorporates RepLKNet (Ding et al., 2022) in the backbone to enhance feature extraction, particularly for severe occlusion and lighting variations. Next, RepNCSPELAN4 (Repeated Normalized Cross Stage Partial with Efficient Large Kernel Attention Network) (Dixit, 2024) is used for multi-scale feature fusion, improving detection across different growth stages and sizes. DynamicHead (Dai et al., 2021) is incorporated as the detection head, using a self-attention mechanism to enhance scale, spatial, and task awareness. Finally, PolyLoss (Leng et al., 2022) is applied to optimize the model’s convergence, improving localization and detection performance for strawberries at various growth stages.

## 3 Materials and methods

### 3.1 YOLOv8 baseline network structure

YOLOv8, released by Ultralytics in 2023, is a next-generation object detection algorithm that integrates state-of-the-art (SOTA) technologies, offering significant improvements in detection speed and accuracy compared to previous versions (Sohan et al., 2024). The YOLOv8 network architecture comprises four key components: Input, Backbone, Neck, and Detection Head. The Input component is responsible for preprocessing images. The Backbone network utilizes a CSPDarknet structure and introduces the C2f module to replace the C3 module in YOLOv5, integrating the Efficient Layer Aggregation Network (ELAN) from YOLOv7 as part of the architecture. This design strengthens feature fusion capabilities by adding cross-layer skip connections and branching structures, while maintaining the network’s lightweight nature (Dong et al., 2023; Wang et al., 2023). The Neck network adopts a Path Aggregation Network-Feature Pyramid Network (PAN-FPN) structure, which combines top-down and bottom-up path aggregation networks. It removes convolution operations after upsampling and replaces the C3 module with the C2f module, achieving a lightweight design while preserving high performance (Guijin et al, n.d.). The Detection Head employs a Decoupled-Head structure that separates the classification and regression tasks into two parallel sub-networks, reducing task conflicts and enhancing the model’s generalization capability and robustness. YOLOv8 also adopts an anchor-free detection strategy, directly predicting the center and boundary information of objects, which significantly improves performance in small object detection, making it highly suitable for various complex real-world applications (Wu and Dong, 2023).

### 3.2 Improved YOLOv8 structure design (RLK-YOLOv8)

**Figure 1:**
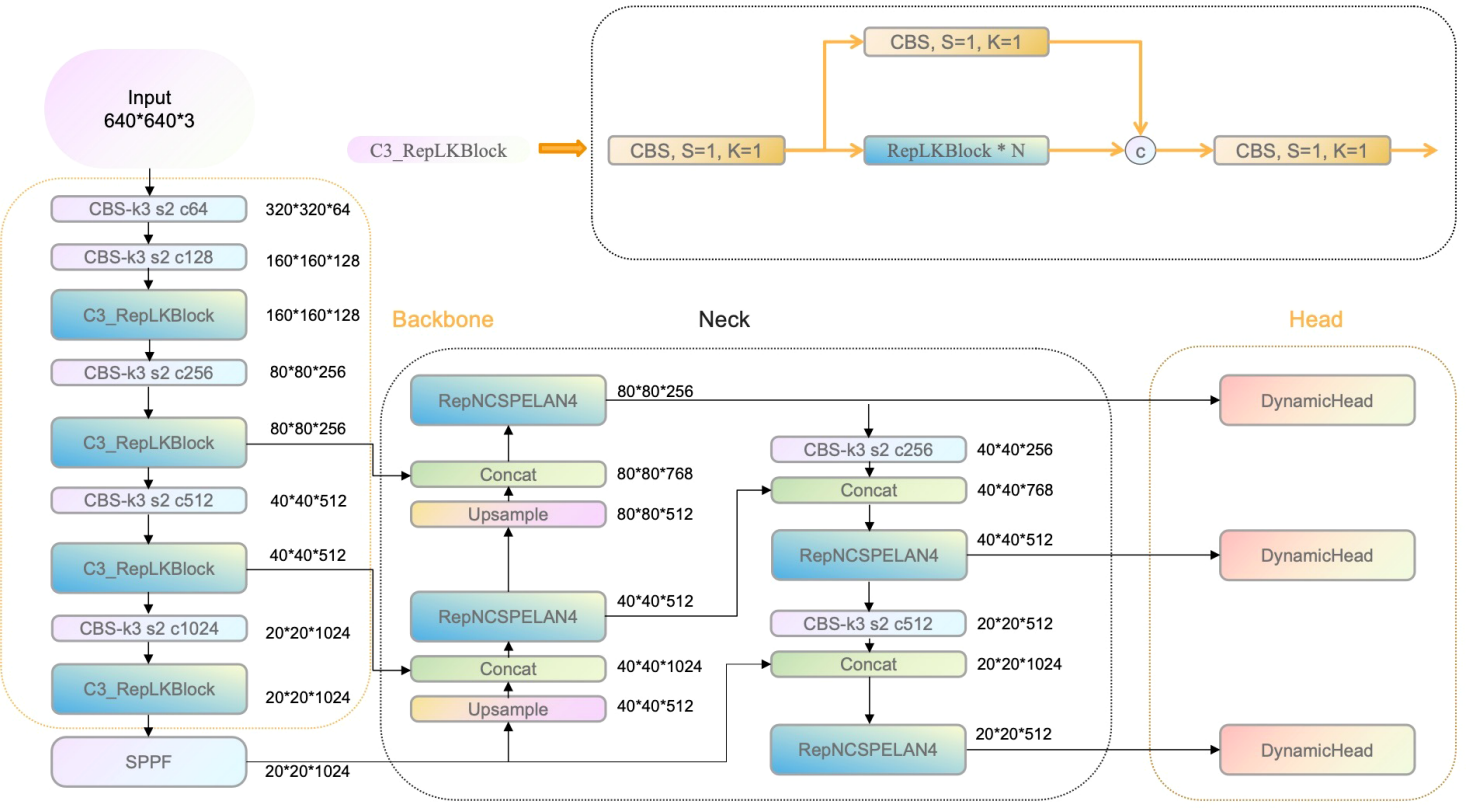
RLK-YOLOv8 Network Architecture Diagram.

To achieve multi-stage detection for greenhouse strawberries throughout their entire growth cycle and address challenges such as feature extraction difficulties and insufficient receptive field in complex environments, this study designs a novel detection algorithm based on the YOLOv8 model (as shown in **Figure 1**). The proposed improvements consist of four modules:

1. C3_RepLKBlock Module (Backbone Part): While the existing C2f module in YOLOv8 extracts features using conventional convolutions, its small kernel size may lose critical information, especially in scenarios where target edges are blurred or mixed with the background. Therefore, the C3_RepLKBlock module is introduced into the YOLOv8 backbone, leveraging large kernel convolutions to boost the model’s capability in capturing complex features of strawberries. This structure effectively retains more edge and detail information in scenarios involving small objects and significant occlusion, thereby ensuring more accurate feature representation at different growth stages of strawberries such as flowering and fruit development.
2. RepNCSPELAN4 Module (Neck Part): This module serves as an improved version of the feature pyramid architecture, incorporating cross-scale feature aggregation and enhanced hierarchical feature fusion. In scenarios with dense multiple targets, it excels in extracting multi-scale information from strawberry fruits, thereby improving detection stability and accuracy.
3. DynamicHead Module (Head Part): The DynamicHead module leverages a multi-level perception strategy combined with a self-attention mechanism to equip the detection head with enhanced scale awareness, spatial awareness, and task awareness. This innovation significantly improves the localization accuracy of bounding boxes, particularly excelling in scenarios with intricate backgrounds and dim lighting. Moreover, the DynamicHead is adept at accommodating variations in target size, thereby enhancing the model’s overall robustness.
4. PolyLoss Module (Loss Part): This loss function optimizes the regression process of bounding boxes by performing precise adjustments to the bounding box positions and shapes using a polynomial formulation. The PolyLoss effectively handles bounding box overlap issues and localization bias, thereby increasing overall detection accuracy and accelerating convergence speed.

#### 3.2.1 Reparameterized large-kernel network (Backbone)

**Figure 2:**
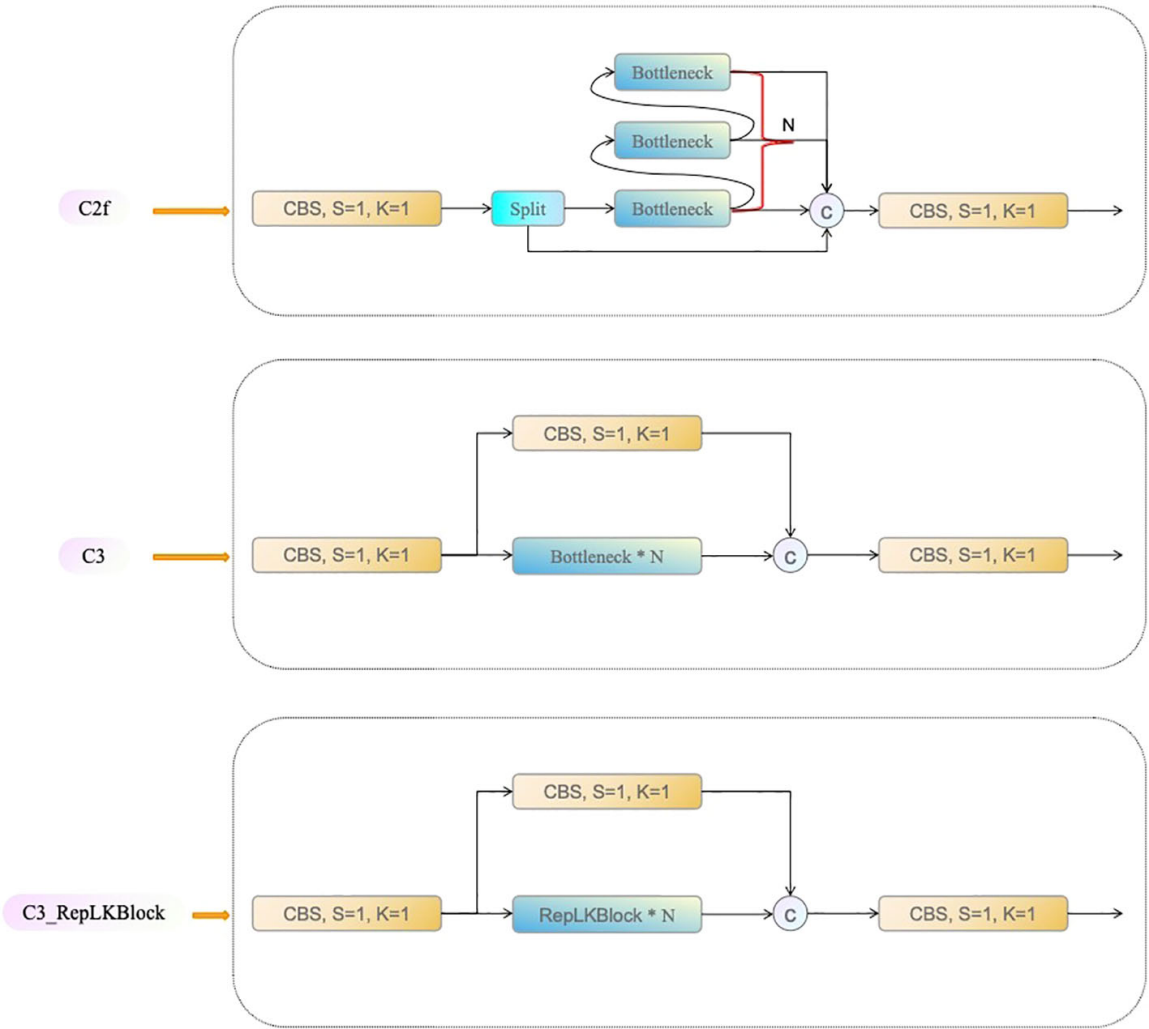
Comparison of Network Structures: C2f, C3, and C3_RepLKBlock.

Compared to traditional small-kernel networks, large-kernel convolutions can more effectively capture global contextual information, which is particularly beneficial in target detection tasks with complex backgrounds (Li et al., 2023). For instance, in downstream tasks such as ImageNet, RepLKNet employs a large 31×31 kernel, which not only achieves comparable performance to the Swin Transformer but also demonstrates lower latency and computational cost (Ding et al., 2024).This study enhances the YOLOv8 architecture to overcome its limitations in receptive field for multi-stage strawberry fruit recognition. YOLOv8 incorporates the C2f module, which processes feature information by splitting the input feature map into multiple branches, with some undergoing bottleneck convolutions before merging. While this design is computationally efficient, it relies primarily on small kernels, limiting its receptive field and making it suboptimal for tasks requiring extensive contextual information or when dealing with complex backgrounds. Consequently, this study proposes replacing the C2f module in YOLOv8 with the novel C3_RepLKBlock component to enhance the model’s feature extraction capability in complex scenes (as shown in **Figure 2**).

**Figure 3:**
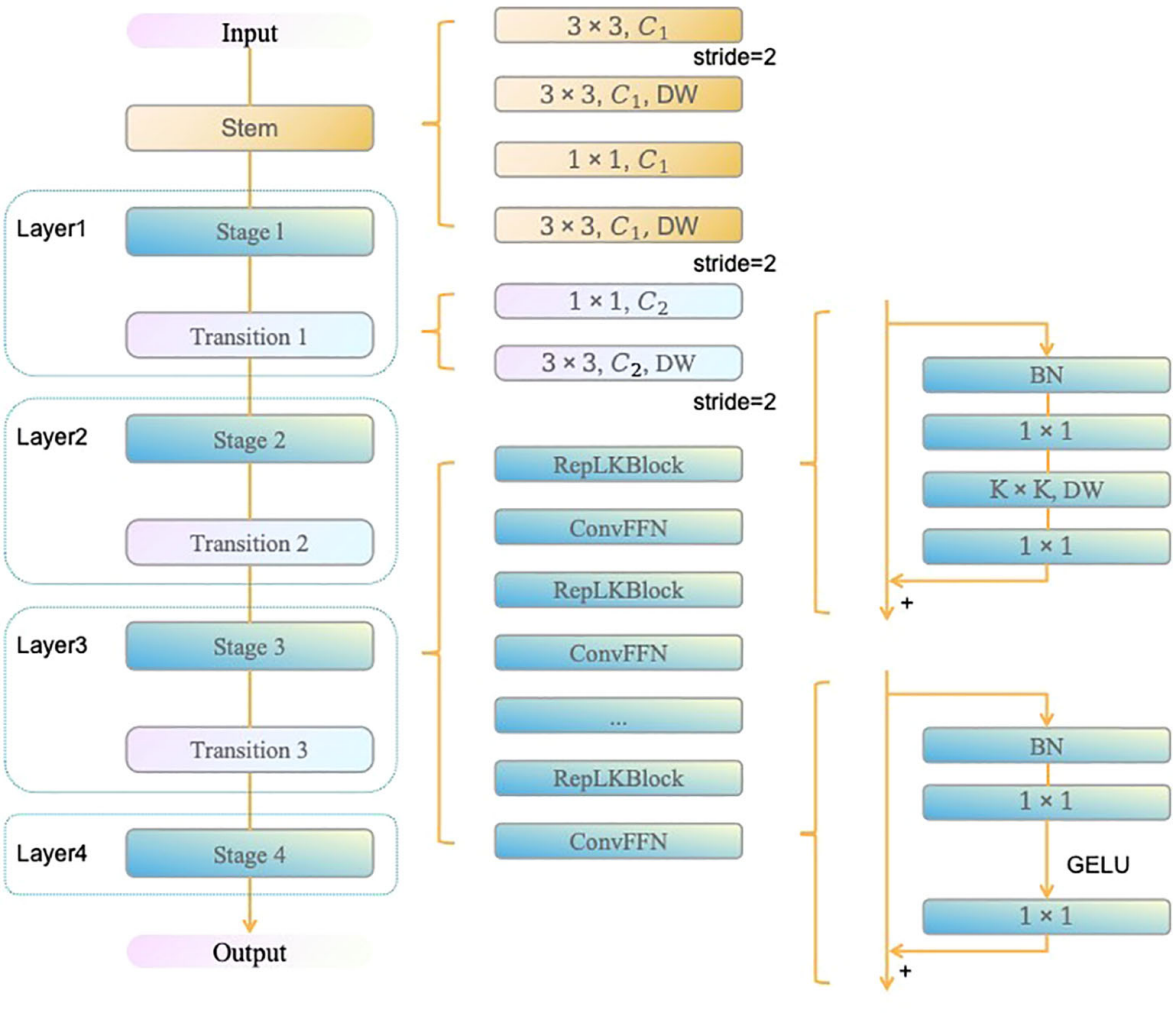
RepLKBlock Network Architecture Diagram.

The C3_RepLKBlock combines the multi-branch processing advantages from the CSP (Cross Stage Partial) structure with the large-kernel design of RepLKBlock (as shown in **Figure 3**), significantly expanding the model’s receptive field. In this module, the input feature map is initially split into two branches via a 1×1 convolution. One branch is subjected to deep convolutions and global feature extraction through multiple layers of RepLKBlock, while the second branch retains the initial input features and is later fused with the first branch’s output through a residual connection. Experimental results show that by introducing a large 27×27 kernel, this module effectively captures global contextual information in greenhouse strawberry scenes, overcoming the limitations of small-kernel networks when handling large targets or complex backgrounds.

In addition, the C3_RepLKBlock integrates the advantages of both small and large convolutional kernels, achieving a balance between capturing global feature and preserving local details, which is particularly effective in multi-scale object detection tasks. This enhancement endows the model with stronger spatial information aggregation capabilities in multi-stage strawberry fruit recognition, resulting in significant improvements in both detection accuracy and efficiency. Compared to the original YOLOv8 architecture, this modification better captures the subtle differences across various growth stages of strawberry fruits, providing more accurate recognition results in complex greenhouse environments.

#### 3.2.2 RepNCSPELAN4 module (Neck)

In complex scenarios, the surface features of greenhouse strawberries are easily affected by background interference, resulting in a less defined boundary between the fruit’s edges and the surrounding background. The existing C2f module in the Neck network primarily utilizes conventional convolution (Conv) for feature extraction from input images. This not only generates a substantial amount of redundant structural information, increasing the computational complexity of the model, but also potentially causes false positives and missed detections. As input data undergoes multiple layers of feature extraction and spatial transformations, there is a loss of original information. This loss may lead the network to establish incorrect associations between the target and the input, thereby skewing the model’s predictions. According to the information bottleneck principle (Tishby, n.d.), input data X passing through consecutive network layers can result in information loss. During backpropagation, this may cause gradient vanishing, which impacts parameter fitting and prediction of Y (as shown in Equation 1).


(1) $$I\left(X,X\right)\geq I\left(Y,X\right)\geq I\left(Y,f_{\theta }\left(X\right)\right)\geq I\left(X,g_{\varphi }\left(f_{\theta }\left(X\right)\right)\right)$$

Where, I represents mutual information, while f and g denote transformation functions with θ and φ as their respective parameters. In a deep neural network, $f_{\theta }\left(\;\right)$ and $g_{\varphi }\left(\;\right)$ correspond to two consecutive layers within the network. According to Equation 1, as the number of network layers increases, the probability of losing original information also grows. Deep neural networks exhibit a decreased ability to preserve comprehensive information about the predicted target at the output stage. Consequently, the use of incomplete information during network training can result in unreliable gradients and poor convergence.

**Figure 4:**
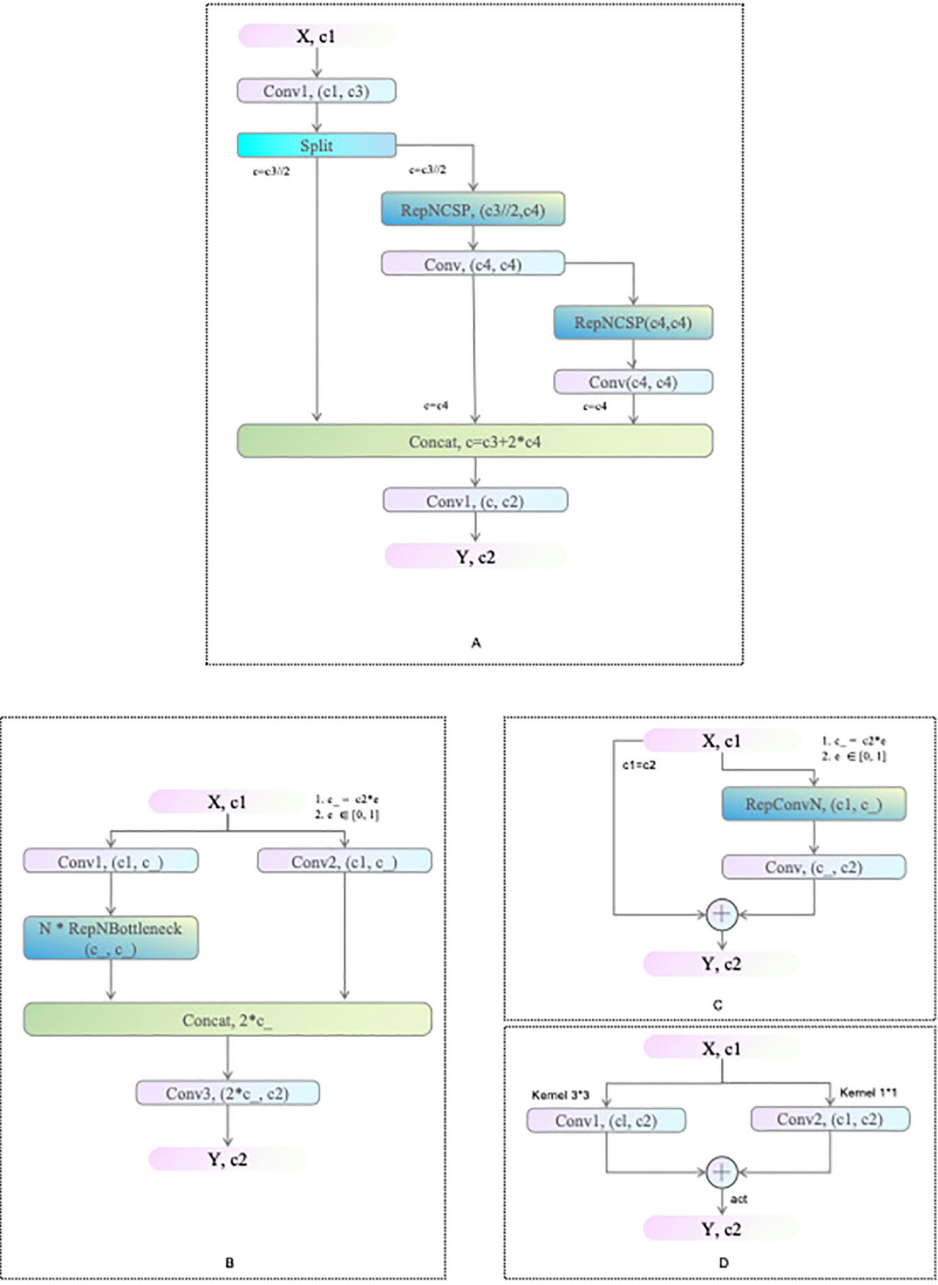
Network Structures of RepNCSPELAN4 **(A)**, RepNCSP **(B)**, RepNBottleneck **(C)**, and RepConvN **(D)** Modules.

RepNCSPELAN4 is an advanced feature extraction and fusion module that ingeniously integrates the strengths of the Cross-Stage Partial Network (CSPNet) and the Efficient Layer Aggregation Network (ELAN) to design an efficient and versatile aggregation network structure (Wang et al., 2024) (as shown in **Figure 4A**). This module primarily composed of a convolution module and RepNCSP, with RepNCSP incorporating convolution modules and several RepNBottleneck submodules (as shown in **Figure 4B**). Serving as the foundational building block, RepNBottleneck integrates reparameterized convolution blocks with the SiLU activation function, significantly enhancing the network’s learning capability (as shown in **Figures 4C, D**). The architecture of this module is shown in **Figure 4**, where X and Y represent the input and output data of the module, respectively, and c1 and c2 indicate the input and output channel numbers, respectively.

**Figure 5:**
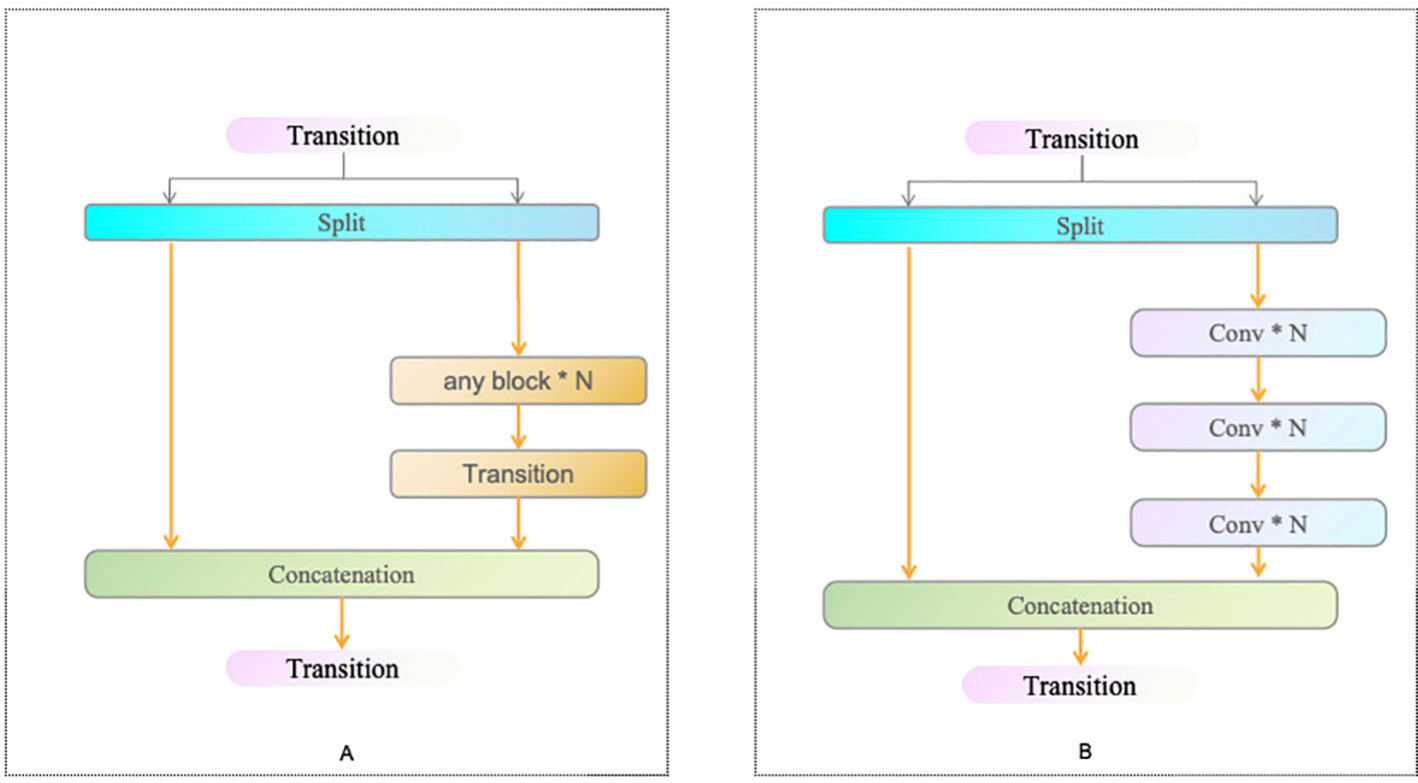
CSPNet **(A)** and ELAN **(B)** Structures.

CSPNet addresses the problem of redundant gradient information by integrating feature maps at different stages of the network, thereby enhancing learning capability while reducing computational cost and memory usage (as shown in **Figure 5A**). Moreover, ELAN ensures the convergence and gradient transmission efficiency of deep networks by designing an efficient gradient propagation path (as shown in **Figure 5B**). Consequently, RepNCSPELAN4 not only improves the model’s feature extraction capabilities but also optimizes computational efficiency, achieving an optimal balance between performance and resource utilization.

In various network layers, the RepNCSPELAN4 module applies upsampling techniques to enhance the spatial resolution of feature maps. By concatenating the upsampled feature maps with those from the preceding layer, the module facilitates multi-scale data integration, thereby preserving fine-grained features and spatial connections, ultimately improving target localization and recognition accuracy.

In YOLOv8, although the C2f module enhances the model’s ability to capture detailed and semantic information, it also increases computational complexity, thus affecting the balance between performance and resource usage. Replacing the C2f module in the Neck with the RepNCSPELAN4 module reduces computational overhead and significantly improve model performance. By addressing the issue of redundant gradient information and utilizing an efficient gradient propagation path, the RepNCSPELAN4 demonstrates excellent performance in balancing model accuracy and computational efficiency.

#### 3.2.3 DynamicHead module (Head)

The main role of the detection head is to handle target recognition and localization. Typical detection heads include the basic detection head and the AsDDet detection head. The basic detection head extracts information from feature maps and outputs bounding box predictions and class probabilities. Although the AsDDet detection head employs an asymmetric structural design, which allows feature maps from the backbone network to be divided into two prediction branches after channel adjustment—thereby decoupling classification and regression tasks—it requires high computational resources and exhibits weak generalization capabilities. Consequently, the AsDDet detection head is not well-suited for multi-stage detection of greenhouse strawberries as a universal detection head.

**Figure 6:**
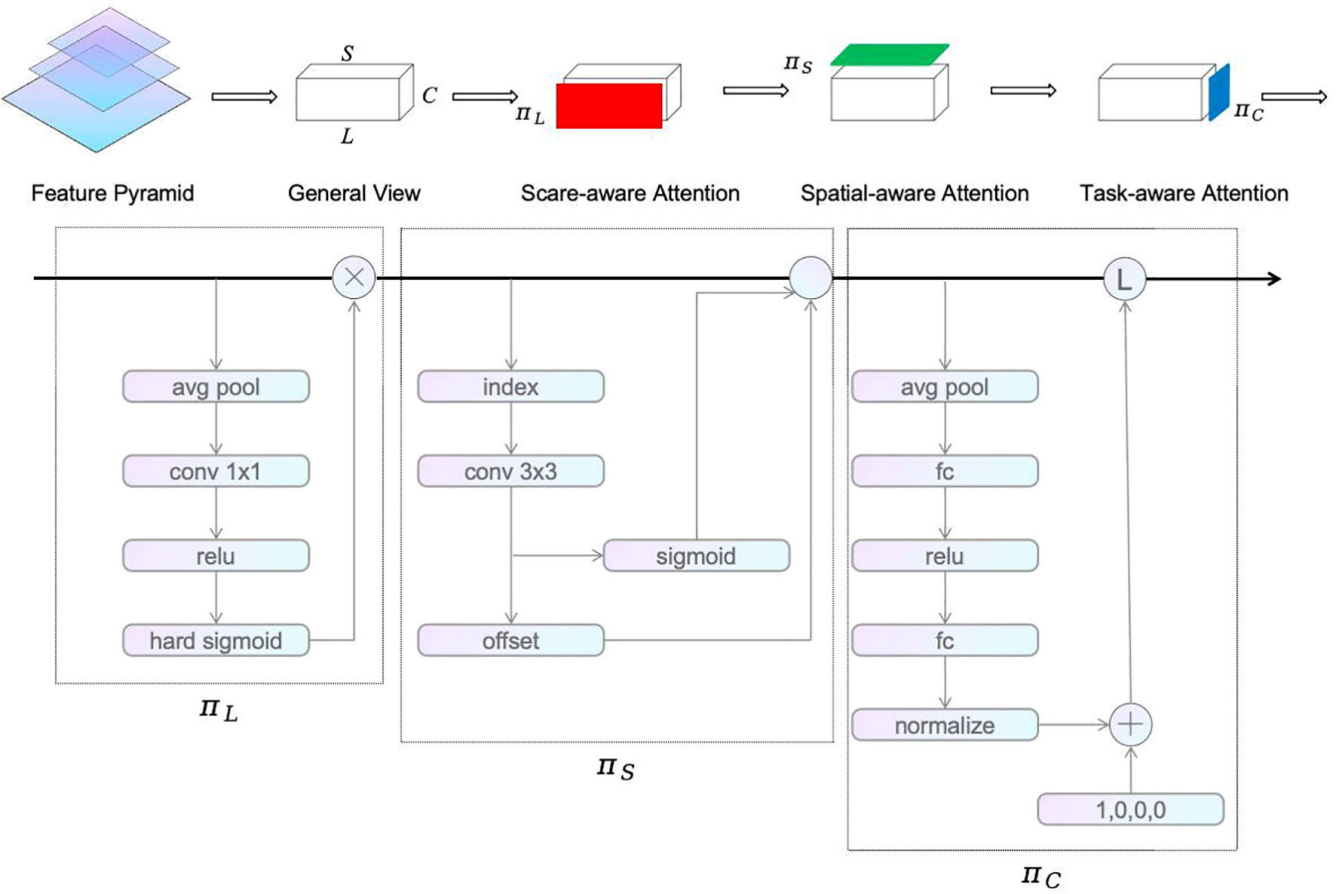
DynamicHead Network Architecture.

To address these issues, this study adopts the DynamicHead detection head to replace the AsDDet detection head, aiming to enhance the model’s detection performance and adaptability. The DynamicHead detection head utilizes attention mechanisms from three perspectives: scale awareness, spatial localization, and multi-task adaptation. The self-attention mechanism between feature hierarchies facilitates the model to more accurately identify targets of varying sizes. The self-attention mechanism across spatial positions allows the model to focus more precisely on specific regions within the image, thereby improving the accuracy of target localization. The self-attention mechanism across output channels enables the model to adjust attention distribution based on the demands of different tasks, thereby enhancing the model’s flexibility and adaptability. The architecture of the DynamicHead is illustrated in **Figure 6**.

Before utilizing DynamicHead, the feature pyramid outputs from the backbone network must be scaled to a consistent resolution. The scaled feature pyramid can be represented as a four-dimensional tensor $F\in R^{L\times H\times W\times C}$ , where L denotes the number of feature pyramid levels, H represents the height of the feature maps, W represents width of the feature maps, and C represents the number of channels (Dai et al., 2021). Further, define S=H×L , conceptualizing the feature map as a three-dimensional tensor with dimensions L , S and C , which correspond to scale awareness, spatial awareness, and task awareness, respectively. The formulas are as follows (as shown in Equation 2):


(2) $$W_{L}\left(F\right)=\pi_{C}\left(\pi_{S}\left(\pi_{L}\left(F\right)\cdot F\right)\cdot F\right)\;$$

(3) $$\pi_{L}\left(F\right)=\sigma \left(f\left(\frac{1}{SC}\sum_{S,C}F\right)\right)\cdot F$$

(4) $$\;\pi_{S}\left(F\right)\cdot F=\frac{1}{L}\sum_{l=1}^{L}\sum_{k=1}^{K}w_{l,k}\cdot F\left(l;P_{k}+\Delta P_{k};c\right)\cdot \Delta m_{k}$$

(5) $$\;\pi_{C}\left(F\right)\cdot F=max\left(\alpha^{1}\left(F\right)\cdot F_{c}+\beta^{1}\left(F\right),\alpha^{2}\left(F\right)\cdot F_{c}+\beta^{2}\left(F\right)\right)\;$$

Where, $W_{L}$ represents the attention function, $\pi_{L}$ denotes the scale-aware attention, $\pi_{S}$ represents the spatial-aware attention, and $\pi_{C}$ refers to the task-aware attention. σ is the Hard Sigmoid function, while f is a linear function similar to a 1×1 convolution (as shown in Equation 3). K indicates the number of sparse sampling locations, $P_{k}+\Delta P_{k}$ is the self-learned spatial offset, $\Delta P_{k}$ represents the ambiguous region. $\Delta m_{k}$ represents the self-learned importance (as shown in Equation 4). $F_{c}$ is the feature of the c-th channel split, α and β are learnable parameters that can be used to control the activation threshold (as shown in Equation 5).

After mapping, the feature maps sequentially pass through the scale-aware module ( $\pi_{L}$ ), spatial-aware module ( $\pi_{S}$ ), and task-aware module ( $\pi_{C}$ ), achieving a unified attention mechanism within the detection head. In the scale-aware module, the dimension of the feature map is first subjected to global average pooling, followed by a 1×1 convolution layer and ReLU activation function for feature extraction. Subsequently, the features are activated by the hard-sigmoid function, and the output is fused with the input feature map to produce the scale-aware module’s output.

In the spatial-aware module, a 3×3 convolution is applied to the input tensor to obtain the feature map’s offset values and weights. Deformable convolution is utilized to further capture positional information of the targets in the feature maps. The task-aware module reduces the parameters by performing global average pooling along the L×S dimension. Subsequently, it refines the features through two fully connected layers followed by a normalization operation, which helps to reduce noise and enhance the stability of feature representation. This two-layer fully connected network efficiently compresses the feature map’s dimensions while highlighting key features. Finally, with the help of a dynamic ReLU activation function, the module further enhances the model’s adaptability across different tasks, achieving precise task awareness. DynamicHead incorporates a multi-head self-attention mechanism in the detection head, enabling the model to flexibly to targets of varying sizes and complexities, thereby improving its flexibility and generalization capability.

#### 3.2.4 PolyLoss module (Loss)

In the optimization of classification models, the choice of the loss function plays a crucial role in determining model performance. The traditional cross-entropy loss function has demonstrated outstanding performance in numerous classification tasks. However, when faced with dataset exhibiting imbalanced class distribution, the model tends to favor classes with larger sample sizes, leading to a decline in generalization ability (Khan et al., 2017). To address this issue, this study introduces the PolyLoss loss function, which is based on the Taylor expansion of the cross-entropy loss function (Leng et al., 2022). By expanding the cross-entropy loss function using a Taylor series and adding perturbations to the first N terms of the expansion, PolyLoss provides a more flexible structure that can be suitable for different tasks and datasets. This flexibility effectively mitigates the influence of data imbalance and enhances the model’s ability to discriminate minority class samples. This study further improves the PolyLoss loss function by incorporating a weight balancing factor and a tuning factor, making the loss function more effective in handling class imbalance between positive and negative samples as well as difficult-to-classify samples. The improved PolyLoss loss function is expressed as Equations 6–7.


(6) $$L_{PolyLoss}\left(p,y\right)=a_{t}\cdot {\left(1-p_{t}\right)}^{\gamma }\cdot BCE\left(p,y\right)+\epsilon \cdot {\left(1-p_{t}\right)}^{\gamma +1}$$

(7) $$a_{t}=y\cdot a+\left(1-y\right)\cdot \left(1-a\right)$$

Where, the Binary Cross-Entropy (BCE) loss function is commonly used in binary classification tasks to measure the difference between the predicted probabilities and the actual labels. Its definition is as Equation 8.


(8) BCE(p,y)= −[y·log(p)+(1−y)·log(1−p)] 

Where, p represents the predicted probability from the model, and y is the actual label (with values of 0 or 1). BCE is typically used in conjunction with the sigmoid function to ensure that the output predictions are probability values. By integrating BCE into the PolyLoss framework, the imbalance issue of samples in binary classification tasks can be effectively addressed.

The improved PolyLoss function significantly enhances the model’s overall performance on imbalanced datasets, particularly in the classification of underrepresented classes, where it shows marked advantages. By integrating pre-trained hidden layer features with residual features and training with the improved PolyLoss loss function, the model demonstrates superior robustness and generalization across various classification tasks. This approach not only effectively resolves the problem of data imbalance but also provides a flexible and efficient loss function option for a wider range of classification scenarios.

## 4 Experiments and results analysis

### 4.1 Data collection and preprocessing

**Figure 7:**
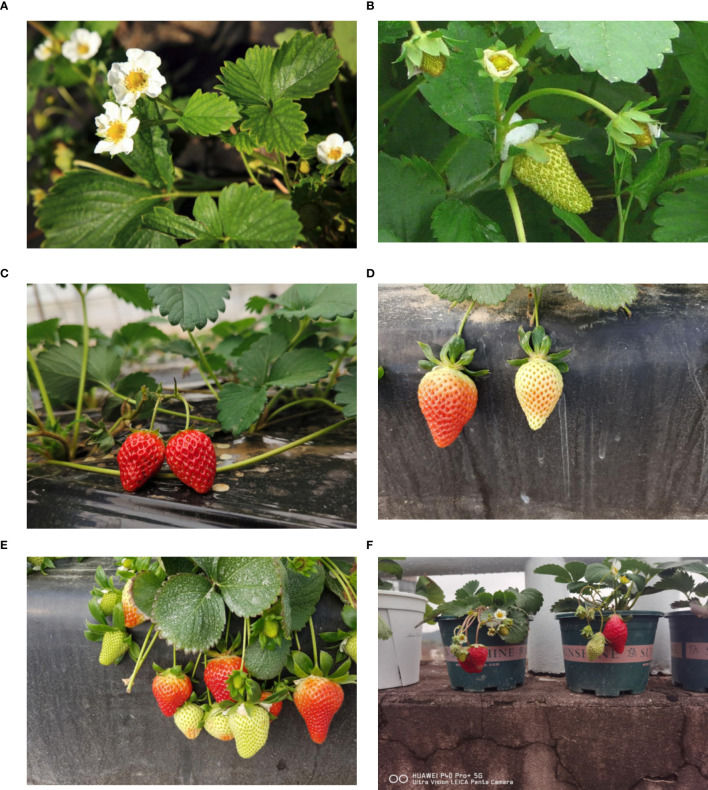
Strawberry Samples in Different Growth Stages and Environments: **(A)** Full Growth Cycle of Strawberries - Flowering Stage, **(B)** Full Growth Cycle of Strawberries - Fruit Development Stage, **(C)** Full Growth Cycle of Strawberries - Fully Ripened Stage, **(D)** Full Growth Cycle of Strawberries - Half-Ripened Stage, **(E)** Full Growth Cycle of Strawberries - Complex Samples, **(F)** Full Growth Cycle of Strawberries - Campus-Grown Samples.

The experimental data were collected from strawberry greenhouse plantations in Lin’an District, Hangzhou, Zhejiang Province, as well as from strawberries cultivated on campus of Zhejiang A&F University. The images were captured using a Huawei P40 Pro+ smartphone. Data collection spanned from mid-October 2023 to mid-April 2024, covering both autumn and winter seasons. The collection took place daily between 1:00 PM and 5:00 PM, with shooting angles ranging from 30°to 60° and shooting distances randomly set. The image’s resolution is 6000×4000 pixels. Ultimately, a total of 3,628 raw images were obtained, with examples shown in **Figure 7**.

**Figure 8:**
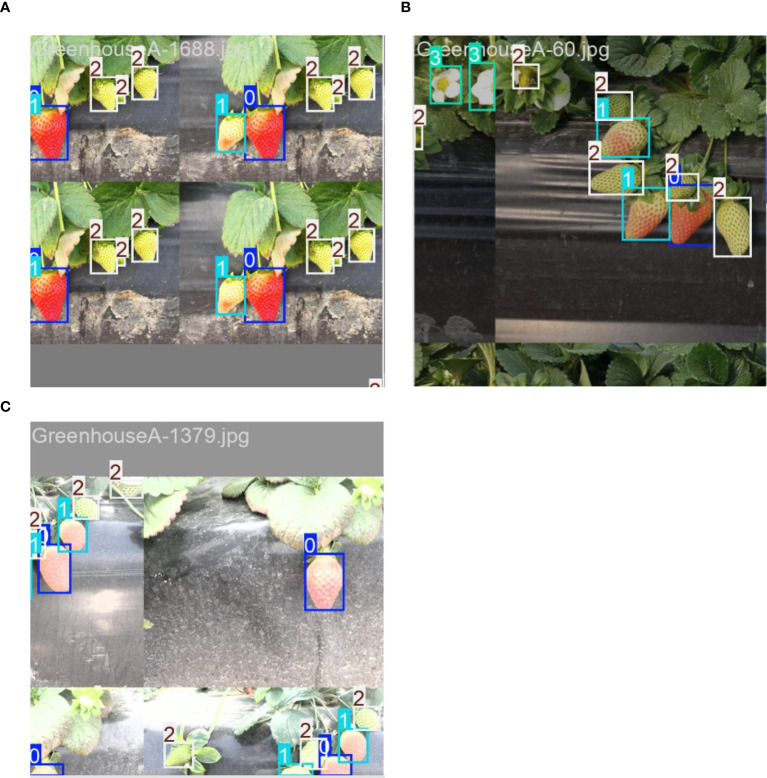
Strawberry Fruits throughout the Full Growth Cycle image’s data enhancement effect: **(A–C)** represent the data enhancement effect after different methods. Use the numbers 0-3 to label the different growth stages of strawberry fruits: 0 = fully-ripened, 1 = half-ripened, 2 = fruit-development, 3 = flowering.

In the training process, we utilized random image scaling and Mosaic data augmentation techniques as online data augmentation methods. The Mosaic data augmentation technique involves randomly scaling four images targeted for detection and seamlessly stitching them into a single novel composite image (as shown in **Figure 8**), followed by occlusion processing in local regions to simulate various complex background environments. This approach not only enriches the background information surrounding the detection targets but also effectively enhances the model’s detection accuracy under challenging scenarios.

**Figure 9:**
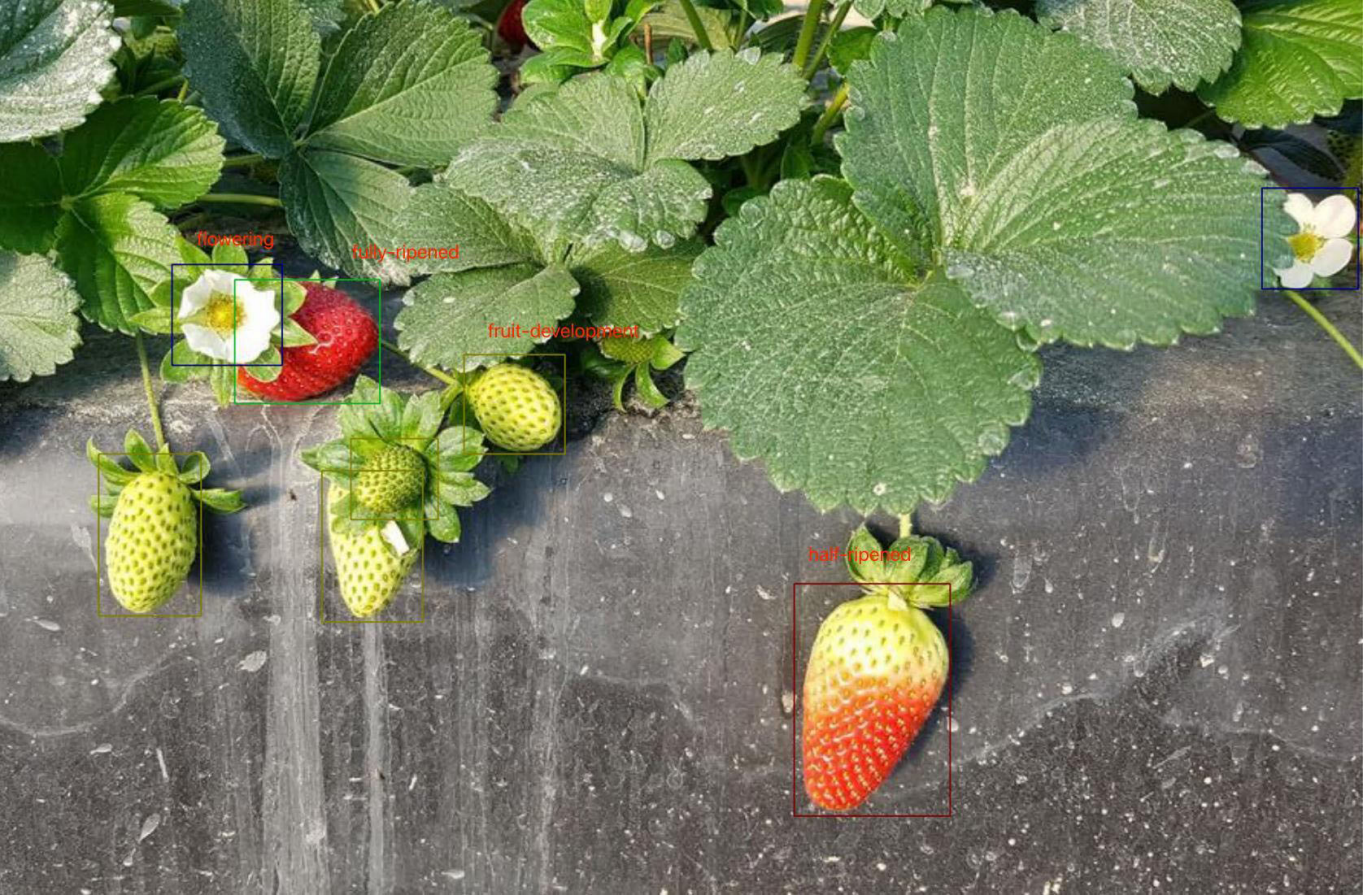
Growth Stage Classification Standards for Strawberries in the Full Growth Cycle.

Subsequently, the dataset was manually annotated using the labeling tool LabelImg, with rectangular bounding boxes used to mark the locations and categories of different stages in the full growth cycle of strawberry fruits. In this study, the categories of strawberry growth stages were divided into four classes (as shown in **Figure 9**): the flowering stage is labeled as “flowering,” the fruit development stage as “fruit-development,” the half-ripened stage as “half-ripened,” and the fully ripened stage as “fully-ripened”.

Considering that strawberries in the image may be severely obscured by surrounding plant parts, affecting recognition accuracy, we excluded strawberry fruits with a surface area exceeding 80% that were obscured from the annotation. The dataset was afterwards randomly divided into training, validation, and testing sets in a ratio of 8:1:1, with the training images independently and uniformly sampled from the entire dataset. The final distribution of the number of images and target objects in the dataset is shown in **Table 1**.

**Table 1:** Dataset for Multi-Stage Strawberry Growth Cycle.

Dataset	Number of images	Flowering Stage	Fruit Development Stage	Half-Ripened Stage	Fully Ripened Stage
Train set	2902	1220	9537	2403	2843
Validation set	362	190	1238	310	328
Test set	364	141	1172	296	316
Total	557	1551	11947	3009	3487

### 4.2 Experimental environment and method

All experiments in this study were conducted under the same hardware environment, with the detailed configuration parameters shown in **Table 2**. A transfer learning training strategy was employed, leveraging the common feature knowledge of convolutional layers to achieve more stable network learning. By combining pretraining and fine-tuning approaches, the generalization ability of the network was significantly improved.

**Table 2:** Experimental Environment.

Configuration	Parameter
CPU	Intel(R) Xeon(R) Gold 6151 CPU @ 3.00GHz
GPU	NVIDIA Tesla T4
OS	Ubuntu 22.04.2 LTS
Framework	pytorch2.4.1+cu121
CUDA	cuda12.1

In the experiments, the input image size was set to 640×640 pixels to balance detection accuracy and computational efficiency. For model optimization, the AdamW optimizer was used with an initial learning rate of 0.01, dynamically adjusted using the cosine annealing learning rate scheduling strategy to ensure smooth model convergence. To accelerate the training process and reduce the risk of overfitting, the momentum factor was set to 0.937, and the weight decay coefficient was set to 0.0005, ensuring stability during weight updates and preventing excessive parameter adjustment. The entire training process lasted for 300 epochs, employing mixed precision (AMP) training to improve computational efficiency and memory utilization.

Training was divided into two stages: frozen and unfrozen. During the frozen stage, the parameters of the initial layers of the backbone network remained unchanged, focusing on optimizing the model’s later layers to reduce memory usage and accelerate training speed of the model. In the unfrozen stage, all layer weights were unlocked, enabling optimization across the entire network to enhance the extraction of target features.

To enhance the model’s generalization ability, various data augmentation strategies were employed, including horizontal flipping (with a probability of 0.5), HSV color space augmentation (Hue set to 0.015, Saturation set to 0.7, and Value set to 0.4), random translation (± 0.1), and random scaling (± 0.5). Additionally, the probability of Mosaic data augmentation was set to 1.0 to improve the model’s robustness to diverse targets. During the validation phase, non-maximum suppression (NMS) was applied with an Intersection over Union (IoU) threshold of 0.7, and the maximum number of detections per image was limited to 300 to ensure precise target selection.

### 4.3 Evaluation metrics

To evaluate the effectiveness of the detection model, this study utilized Precision, Recall, F1-score, and mean Average Precision (mAP) as evaluation metrics for the multi-stage recognition of greenhouse strawberries throughout their full growth cycle. The F1-score is defined as the harmonic mean of Precision and Recall. The specific definitions are as follows (as shown in Equations 8–10):


(8) $$\begin{array}{c} \;precision=\frac{TP}{TP+FP} \end{array}$$

(9) $$\begin{array}{c} \;Recall=\frac{TP}{TP+FN} \end{array}$$

(10) $$\begin{array}{c} \;F1=2\times \frac{precision\times Recall}{precision+Recall}\; \end{array}$$

(11) $$mAP=\frac{1}{N}\sum_{i=1}^{N}AP_{i}$$

In this context, TP (True Positive) represents the number of strawberries correctly predicted as present, FP (False Positive) refers to the number of instances incorrectly predicted as strawberries, and FN (False Negative) denotes the number of undetected strawberries, i.e., missed detections. The Average Precision (AP) is defined as the area under the Precision-Recall curve, which evaluates the detection performance for a single category. For multi-class recognition tasks, the mean Average Precision (mAP), which is the average of AP across all categories, is used to measure the overall detection accuracy. Here, N represents the total number of categories, and $AP_{i}$ is the average precision for the i -th category. A higher mAP value indicates better overall detection performance of the multi-stage recognition model for greenhouse strawberries throughout their full growth cycle (as shown in Equation 11).

### 4.4 Analysis of experimental results

**Figure 10:**
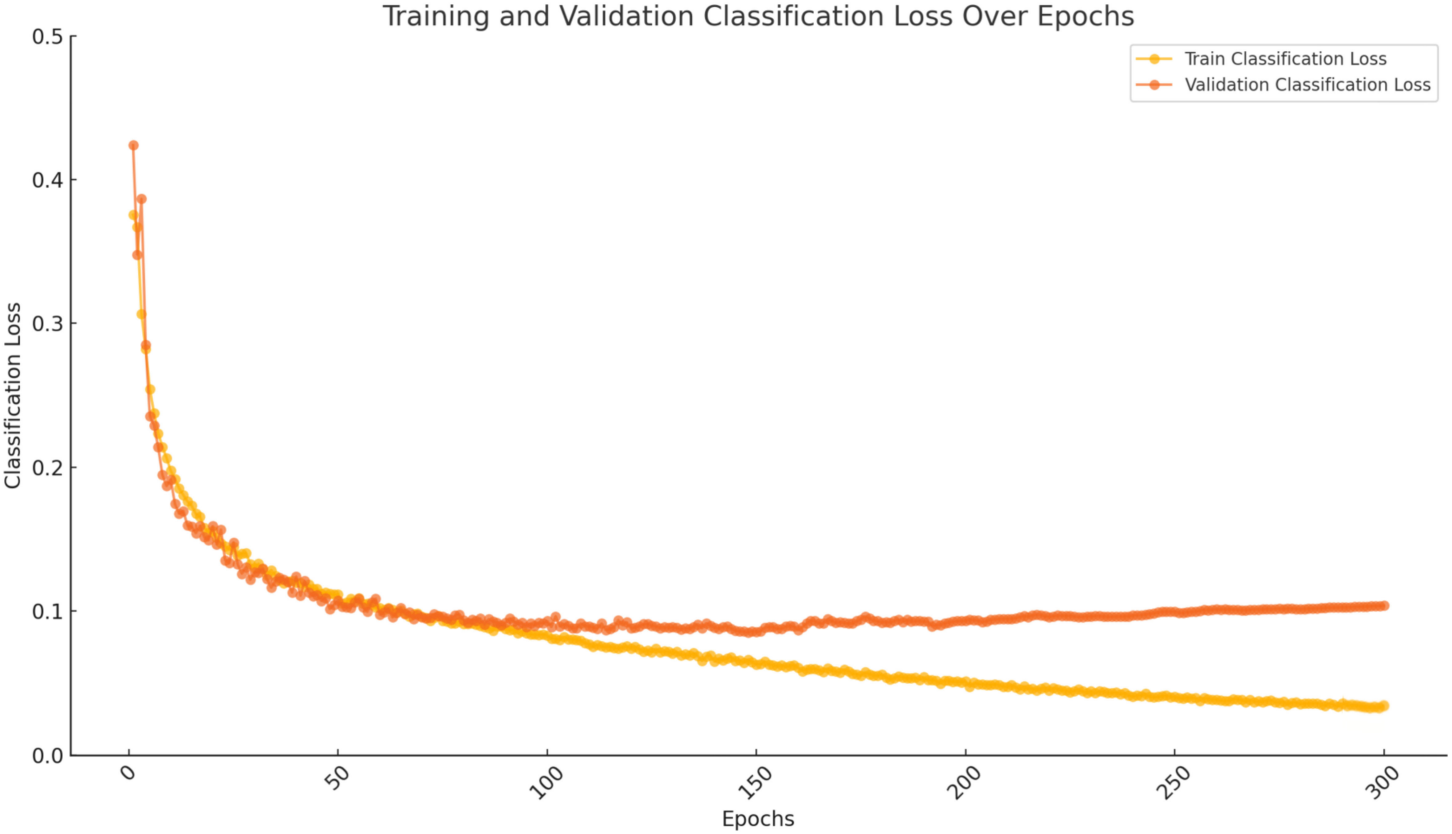
Convergence curve for training dataset and validation dataset.

The model underwent training for 300 epochs. The loss curve of the improved YOLOv8 network showed a sharp decline in loss values within the initial 10 epochs, indicating a rapid model convergence. Subsequently, both the training and validation loss values gradually converged, with the training loss fluctuating between 0.02 and 0.1, and the validation loss ranging from 0.1 to 0.16. As shown in **Figure 10**, the RLK-YOLOv8 model’s training and validation loss values decreased swiftly and tended to stabilize, demonstrating the effectiveness and robust learning capability of the improved model.

Based on the experimental results presented in **Table 3**, RLK-YOLOv8 outperformed the original YOLOv8 in detecting strawberries at different growth stages. For overall detection, RLK-YOLOv8 achieved a 3.3% improvement in precision, reaching 0.934, while the mAP increased from 0.948 to 0.954. Specifically, at different growth stages, RLK-YOLOv8 demonstrated a significant 6.4% increase in precision during the flowering stage and improvements of 1.9% and 2.4% in the fruit development stage and fully ripened stage, respectively. Although RLK-YOLOv8 showed a slight decline in recall during the flowering, fruit development, and fully ripened stages, it remained relatively stable in the half-ripened stage. Furthermore, compared to the original YOLOv8 model, RLK-YOLOv8 achieved higher mAP values across all stages except for a slight decrease in the fruit development stage. Notably, in the half-ripened stage, RLK-YOLOv8 outperformed the original YOLOv8 by 0.9% in terms of mAP. Overall, RLK-YOLOv8 demonstrated more accurate detection of strawberries at different growth stages and exhibited superior comprehensive performance.

**Table 3:** Experimental Results Comparison: RLK-YOLOv8 vs. Original YOLOv8.

Metrics	Stage	YOLOv8	RLK-YOLOv8
Precision	all	0.901	0.934
	flowering	0.887	0.951
	fruit-development	0.932	0.951
	half-ripened	0.856	0.878
	fully-ripened	0.93	0.954
Recall	all	0.896	0.875
	flowering	0.824	0.817
	fruit-development	0.93	0.887
	half-ripened	0.918	0.916
	fully-ripened	0.912	0.878
mAP	all	0.948	0.954
	flowering	0.919	0.932
	fruit-development	0.962	0.959
	half-ripened	0.943	0.952
	fully-ripened	0.966	0.972

To validate the effectiveness of the proposed RLK-YOLOv8 improvements on model performance, we conducted a step-by-step evaluation by progressively integrating various modules into the YOLOv8 framework. Ablation experiments were performed to analyze the feasibility of each improvement strategy, where “√” indicates the application of a corresponding optimization strategy, while “-” denoted its absence. All other training parameters were held constant to ensure the reliability of our results. The Ablation study aimed to reveal the individual contributions of different strategies to model performance. The results, presented in **Table 4**, underscore the significant enhancements in Precision achieved by all improvement strategies. Overall, our findings robustly demonstrate the efficacy of the proposed RLK-YOLOv8 improvements in boosting model performance.

**Table 4:** Ablation Study Results.

Model	C3_RepLKBlock	RepNCSPELAN4	DynamicHead	PolyLoss	Precision	Recall	mAP	F1	FPS
YOLOv8	–	–	–	–	0.901	0.896	0.948	0.8975	45.38
Strategy1	✓	–	–	–	0.928	0.865	0.946	0.895	37.66
Strategy2	–	✓	–	–	0.925	0.894	0.953	0.909	28.68
Strategy3	–	–	✓	–	0.918	0.858	0.94	0.887	41.04
Strategy4	–	–	–	✓	0.913	0.856	0.942	0.884	41.05
OURS	✓	✓	✓	✓	0.934	0.875	0.954	0.903	33

The ‘✓’ symbol indicates that the corresponding optimization strategy was applied, while ‘-’ indicates that it was not.

During the feature extraction process, the C3_RepLKBlock module was introduced to enhance the model’s ability to capture small object features. This improvement demonstrated outstanding performance in Precision (from 0.901 to 0.928), but showed slight declines in mAP (0.948 to 0.946) and F1 (0.8975 to 0.895) compared to the baseline model, indicating areas for improvement in specific scenarios. After incorporating the RepNCSPELAN4 module, the model achieved significant improvements in Precision (0.925), mAP (0.953), and F1 (0.909), highlighting its advantages in handling multi-scale feature fusion and tackling complex object detection tasks. The inclusion of the DynamicHead module effectively enhanced feature aggregation capabilities, leading to a high detection accuracy in more complex tasks, with Precision reaching a high of 0.918. However, Recall and mAP showed slight declines compared to the baseline model, reflecting the trade-off between improving Precision and maintaining other metrics in complex detection tasks. Furthermore, the introduction of PolyLoss exhibited significantly improved bounding box regression, resulting in enhanced Precision and more accurate bounding box positioning.

The final model, RLK-YOLOv8 (OURS), achieved significant comprehensive performance improvements through the integration of these modules. Compared to the original YOLOv8, RLK-YOLOv8 increased Precision to 0.934 and F1 to 0.903, while remaining a nearly unchanged mAP and a compact parameter size of 11.1 MB. Consequently, these improvements not only significantly enhanced detection accuracy but also demonstrated advantages in real-time performance and lightweight characteristics. This makes RLK-YOLOv8 particularly well-suited for multi-stage detection tasks of strawberries throughout the entire growth cycle, especially in complex environments characterized by varying lighting conditions, occlusion, and background interference.

**Figure 11:**
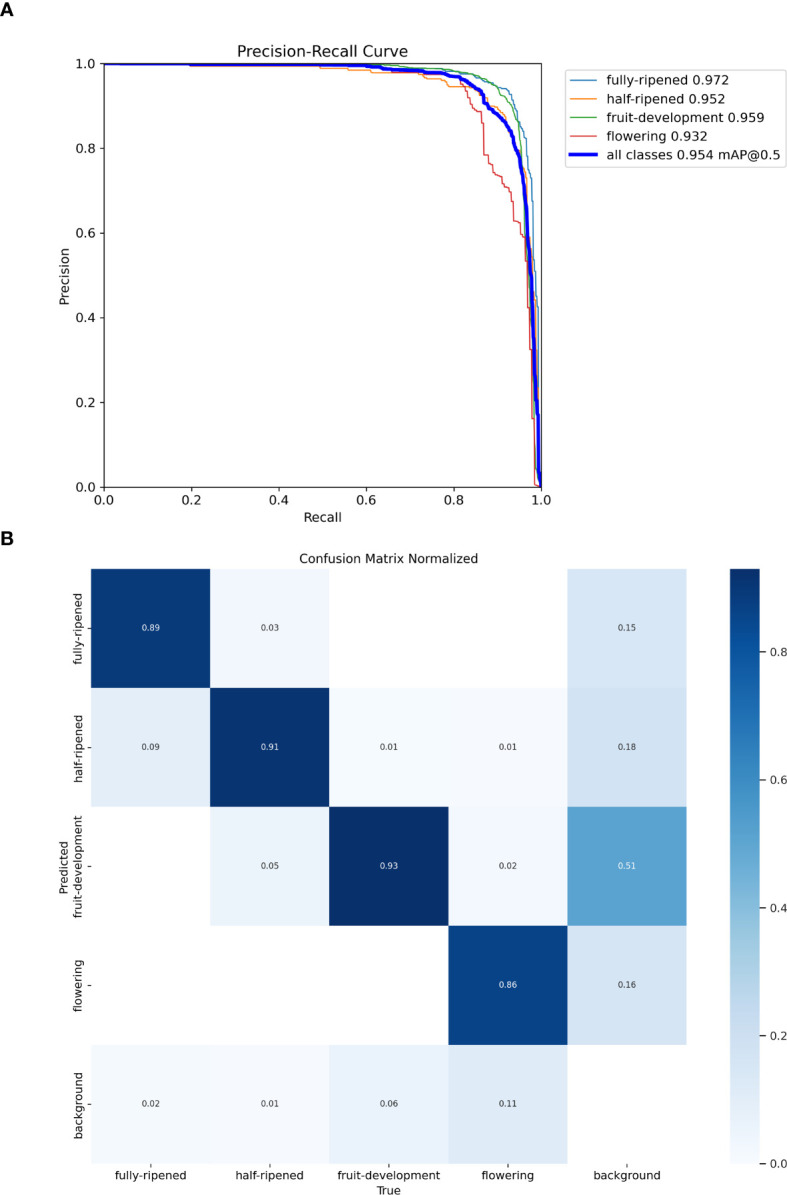
Precision–Recall curve **(A)**: The horizontal axis represents recall, and the vertical axis represents precision. Confusion matrix **(B)**: “Fully-ripened” represents the fully-ripened stage, “Half-ripened” represents the half-ripened stage, “Fruit-development” represents the fruit development stage, “Flowering” represents the flowering stage, and “Background” represents the background class. The rows represent the true labels, and the columns represent the predicted classes.

The precision-recall curve shown in **Figure 11A** demonstrates strong performance across all strawberry growth stages, with the fully-ripened stage achieving the highest precision of 97.2%. The normalized confusion matrix in **Figure 11B** shows high true positive (TP) rates for all stages: 89% for fully-ripened, 91% for half-ripened, and 93% for fruit-development, indicating excellent classification accuracy. The false positive (FP) rate is minimal, and although false negatives (FN) occur slightly more in the flowering stage, the model still performs consistently well. These results highlight the robustness of the RLK-YOLOv8 model in accurately detecting strawberries in complex, occluded environments, making it a promising solution for yield estimation.

**Figure 12:**
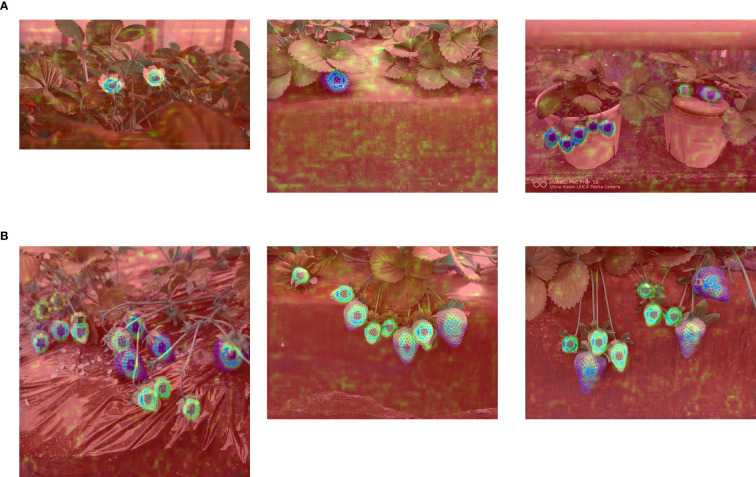
Heatmap Visualizations Based on RLK-YOLOv8: **(A)** Heatmap visualization for strawberry fruit targets with fewer objects. **(B)** Heatmap visualization for strawberry fruit targets with more objects.

Using feature map visualization techniques, heatmap analysis was conducted on greenhouse strawberry fruit images, as shown in **Figure 12**. By extracting the feature maps outputted by the model, and subsequently applying dimensionality reduction, normalization, and pseudo-color mapping, detailed heatmaps of strawberry fruits were generated. Addressing the challenges of small-scale targets and occlusion in strawberry fruits, the RLK-YOLOv8 model demonstrated the ability to accurately focus on the target regions while effectively reducing interference from the background. This approach not only effectively suppressed background noise but also significantly enhanced attention to the target regions, thereby validating the model’s applicability and robustness in the intricate multi-stage detection tasks of greenhouse strawberry fruits across their entire growth cycle.

### 4.5 Comparison with classical detection algorithms

To further assess the effectiveness of the proposed RLK-YOLOv8 model in multi-stage recognition of greenhouse strawberries throughout their full growth cycle, we compared it with mainstream object detection models, including SSD, Faster R-CNN, YOLOv5, YOLOx, YOLOv7, YOLOv8, and the recently released YOLOv9, YOLOv10, and YOLOv11. All experiments were conducted on the same strawberry dataset for training, validation, and testing to ensure the fairness and comparability of the results.

The comparative experiments comprehensively evaluated the performance of different object detection algorithms using seven evaluation metrics: Precision (P), Recall (R), mean Average Precision (mAP), F1-score, Frames Per Second (FPS), Floating Point Operations (FLOPs), and parameter count. These metrics are summarized in **Table 5**.

**Table 5:** Performance Comparison of Different Detection Models.

Model	P	R	mAP	F1	FPS	FLOPs(G)	Parameters(MB)
Faster R-CNN	70.11%	92.85%	92.49%	0.8	5.79	369.793	136.75
SSD	89.69%	76.29%	89.43%	0.82	15.42	61.055	24.013
YOLOv5	91.28%	86.65%	91.72%	0.89	19.37	108.279	46.154
YOLOx	90.64%	92.28%	94.76%	0.92	13.96	155.684	54.15
YOLOv7	91.71%	92.62%	93.95%	0.92	10.66	105.165	37.211
YOLOv8	90.10%	88.31%	93.30%	0.8975	45.38	28.653	11.137
YOLOv9	89.40%	87.60%	94.60%	0.88	22.74	6.702	1.766
YOLOv10	89.20%	88.80%	94.20%	0.89	25.36	8.399	2.709
YOLOv11	90.60%	89.20%	94.90%	0.899	24.44	6.444	2.591
RLK-YOLOv8	93.40%	87.50%	95.40%	0.903	33	50.9	11.1

Two-stage detection algorithm, Faster R-CNN, although demonstrating excellent performance in recall and mAP, struggles to meet the requirements of real-time detection applications due to its high computational cost and parameter size (369.793 GFLOPs and 136.75 MB, respectively). Meanwhile, SSD, while having a lower computational cost (61.055 GFLOPs), exhibits relatively low precision (89.69%) and recall (76.29%), which limiting its applicability in fine-grained detection tasks.

Among the YOLO series models, YOLOv5, YOLOv7, and YOLOx demonstrate strong overall performance, with YOLOx standing out in terms of mAP (94.76%) and recall (92.28%). However, these models have relatively high computational cost (105.165–155.684 GFLOPs) and parameter size (37.211–54.15 MB), which could present limitations on embedded devices. YOLOv8 achieves stable performance in precision (90.10%) and F1 score (0.8975), while maintaining a relatively low parameter size (11.137 MB) and moderate computational cost (45.38 GFLOPs), making it a suitable foundation for further optimization.

YOLOv9, YOLOv10, and YOLOv11, released in 2024, focus primarily on lightweight design and accuracy optimization. YOLOv9 excels in inference speed (FPS) and lightweight design, with the lowest computational cost (6.702 GFLOPs) and a reduced parameter size of 1.766 MB, making it well-suited for low-resource environments. However, YOLOv9 exhibits slightly weaker performance in precision (89.40%) and recall (87.60%), limiting its applicability to high-precision detection tasks. Although YOLOv10 and YOLOv11 excel in their lightweight design and optimal parameter sizes, their inference speed is significantly lower than that of YOLOv8, which may restrict their deployment on embedded devices requiring real-time detection capabilities.

Based on YOLOv8, our proposed RLK-YOLOv8 model demonstrates substantial enhancements in precision (93.40%), mAP (95.40%), and F1 score (0.903). Furthermore, with an inference speed of 33 FPS, it satisfies the needs of typical video recording applications. RLK-YOLOv8 also strikes a balance between performance and efficiency, featuring a moderate computational cost of 50.9 GFLOPs and a compact parameter size of 11.1 MB, making it highly suitable for deployment in embedded and resource-constrained environments.

**Figure 13:**
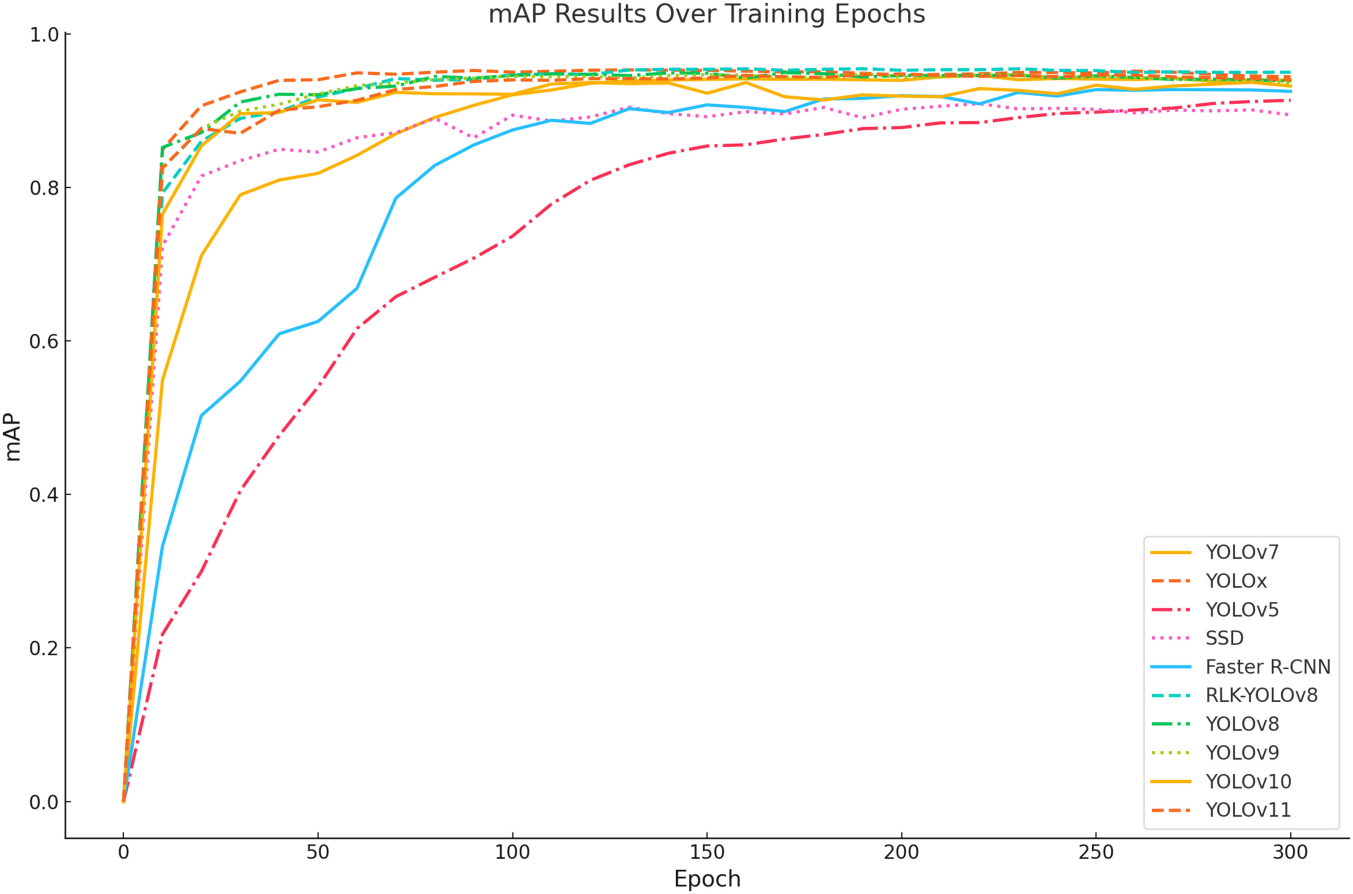
Variation curves of mAP for different models during training. The figure illustrates the mAP performance over training epochs for various models, including YOLOv7, YOLOx, YOLOv5, SSD, Faster R-CNN, YOLOv8, RLK-YOLOv8, YOLOv9, YOLOv10, and YOLOv11. The curves show the mAP values as the models progress through different training epochs, with RLK-YOLOv8 demonstrating notable performance improvements over other models in strawberry detection tasks. The legend identifies the corresponding models for each line.

By examining the trends of mean Average Precision (mAP) across training epochs for multiple object detection models, we present a more intuitive performance evolution through the curve graph (as shown in **Figure 13**). The x-axis denotes the number of training epochs, spanning from 0 to 300, while the y-axis represents the mAP values, ranging from 0 to 1. As depicted in the figure, the mAP curves for different models exhibit a sharp increase during the early stages of training, followed by gradual stabilization. Notably, the mAP curve of RLK-YOLOv8 consistently maintains a higher level compared to the others throughout the entire training process, ultimately achieving the highest mAP value in the final epochs. This demonstrates the superior detection performance of the RLK-YOLOv8 model over the other models analyzed.

In the overall comparison, the trends of mAP for each model across training epochs exhibit significant differences. Both RLK-YOLOv8 and YOLOv8 demonstrate a rapid convergence rate, achieving mAP values exceeding 90% within the first 50 epochs and maintaining high precision stability during subsequent training stages. The mAP performance of RLK-YOLOv8 is slightly superior to that of YOLOv8, suggesting that the enhanced model excels in high-precision object detection tasks. YOLOx also achieves mAP values nearing 90%, with good convergence characteristics, but its performance in later training stages is slightly inferior to that of RLK-YOLOv8 and YOLOv8. In contrast, the mAP curve of YOLOv7 is relatively steady but achieves a lower final accuracy, typically ranging between 80% and 90%. YOLOv5 and SSD exhibit slower convergence rates, with mAP values gradually increasing as training progresses and eventually stabilizing around 80%. Faster R-CNN, however, shows poor convergence, with mAP fluctuating around 70% throughout the training process and failing to reach high accuracy levels.

In summary, RLK-YOLOv8 excels in both final mAP accuracy and convergence speed, demonstrating strong detection capabilities and outstanding generalization performance. These findings indicate that, through the integration of optimization strategies, RLK-YOLOv8 achieves superior detection accuracy in multi-object and complex environments, thereby validating the effectiveness and robustness of its improvements.

**Figure 14:**
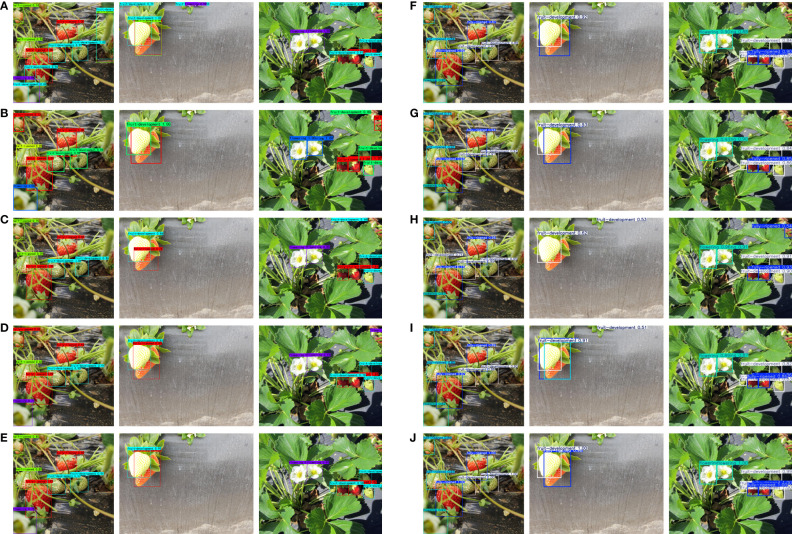
Detection performance comparison of various models under different occlusion conditions: **(A)** Faster R-CNN; **(B)** SSD; **(C)** YOLOv5; **(D)** YOLOx; **(E)** YOLOv7; **(F)** YOLOv8; **(G)** YOLOv9; **(H)** YOLOv10; **(I)** YOLOv11; **(J)** RLK-YOLOv8. (A1–J1): Detection in high target density scenarios with vine occlusion. (A2–J2): Detection in low target density scenarios with mutual occlusion among fruits. (A3–J3): Detection in high target density scenarios with leaf occlusion.

**Figure 14** presents a comparison of prediction results among various network models under actual strawberry growth conditions, highlighting significant differences in detection performance across varying target densities. The detailed analysis is outlined below:

Column 1 (**Figures 14**, A1–J1): In scenarios with high target density and vine occlusion, the detection performance of the models varies notably. Specifically, Faster R-CNN exhibits false positives in the upper right corner and duplicate detections in the lower left corner, while SSD and YOLOx each show one false positive in the upper left corner. YOLOv5 detects some targets but fails to capture certain objects at the edge of the image in the lower left corner. YOLOv10 incorrectly identifies one object on the left side as a positive. In contrast, YOLOv7, YOLOv8, YOLOv9, YOLOv11, and RLK-YOLOv8 demonstrate stable detection under this condition, accurately recognizing all targets and precisely locating bounding boxes without any missed or false detections.

Column 2 (**Figures 14**, A2–J2): In scenarios characterized by low target density but mutual occlusion among fruits, several models exhibit suboptimal detection results. Specifically, Faster R-CNN shows one duplicated annotation and two false positives within its detection boxes. YOLOv5 displays significant localization errors in this context. YOLOv10 experiences missed detections, while YOLOv11 shows false positives and overlapping bounding boxes. Conversely, SSD, YOLOx, YOLOv7, YOLOv8, and RLK-YOLOv8 maintain relatively stable detection accuracy under these conditions, showcasing superior performance with precise bounding boxes and no instances of offset or false detections.

Column 3 (**Figures 14**, A3–J3): In scenarios with high target density and leaf occlusion, the performance differences among models are particularly evident. Faster R-CNN, SSD, YOLOv5, YOLOx, and YOLOv10 all exhibit 1–2 false positives in the upper right corner during detecting dense targets. Notably, SSD, YOLOv5, and YOLOv10 additionally fail to accurately identify one occluded target located in the center. In contrast, YOLOv7, YOLOv8, YOLOv9, YOLOv11, and RLK-YOLOv8 excel in this scenario, achieving precise and error-free bounding box localization for all targets without any overlaps or false detections.

Comprehensive Evaluation: RLK-YOLOv8 demonstrates the best detection performance under various target densities and occlusion conditions, especially in complex scenarios with high target density. It consistently and accurately recognizes and localizes targets. Compared with other models, RLK-YOLOv8 exhibits significant advantages in terms of both detection accuracy and bounding box precision. Furthermore, RLK-YOLOv8 maintains a balance between high detection accuracy and lightweight characteristics, achieving an excellent trade-off between accuracy and real-time performance. Consequently, the RLK-YOLOv8 model provides effectively support for multi-stage detection throughout the entire growth cycle of greenhouse strawberries.

## 5 Discussion

The detection of strawberries throughout their full growth cycle, particularly in greenhouse environments, has long been challenging due to factors like small fruit size, dense packing, occlusion by leaves, and complex backgrounds. Traditional methods of yield estimation often struggle to detect strawberries in such conditions, and many existing algorithms like YOLOv8, while effective in certain applications, still face difficulties when handling multi-target scenarios, occlusion, and varying environmental factors. Similarly, ensemble learning methods have been widely used to improve model robustness in complex prediction tasks. By combining multiple learning techniques, these approaches enhance the performance of the final model, allowing it to generalize better in unpredictable environments (Mishra, n.d.). In this study, we also employed multi-stage detection strategies to increase the precision and recall for strawberry detection under varying greenhouse conditions.

RLK-YOLOv8 addresses these challenges by integrating large kernel convolutions through RepLKNet, enabling more effective feature extraction in dense environments. The multi-scale feature fusion in the RepNCSPELAN4 module further improves the model’s performance, especially in detecting strawberries at various growth stages. The DynamicHead module dynamically adjusts the model’s attention across various scales and occlusions, improving its ability to detect strawberries in complex, overlapping scenarios. These improvements have resulted in a 3.3% increase in precision and a 0.6% improvement in mAP, outperforming YOLOv8 in multi-stage strawberry detection tasks.

Despite these advancements, RLK-YOLOv8 still faces challenges in extreme occlusion and highly overlapping targets. These issues are common in real-world strawberry cultivation, where fruits are densely packed and often partially hidden. To further improve detection, future research could explore multi-sensor fusion and advanced feature extraction techniques, which would help the model better handle occlusion and complex background interference. Additionally, expanding the model’s dataset to include a greater diversity of strawberry varieties and growth conditions will improve its generalization capabilities, enhancing its applicability across different agricultural settings.

## 6 Conclusions

With the rapid development of agricultural digitization and intelligence, the strawberry industry is evolving from traditional manual practices to automated, intelligent harvesting and precision management. This study introduces RLK-YOLOv8, an enhanced YOLOv8 model specifically tailored for greenhouse strawberry detection. Compared to the baseline, RLK-YOLOv8 boosts detection accuracy by 3.3% under challenging conditions, such as multi-object and complex backgrounds, achieving a mean Average Precision (mAP) of 0.954, and an F1-score of 0.903. Its inference speed of 33 FPS and compact parameter size of 11.1 MB make it highly suitable for real-time applications and efficient deployment on embedded and edge devices.

By integrating advanced techniques such as RepLKNet, RepNCSPELAN4, DynamicHead, and PolyLoss, RLK-YOLOv8 significantly enhances feature extraction, multi-scale target detection, and bounding box regression accuracy. These improvements not only validate the superior performance of RLK-YOLOv8 in image detection tasks but also pave the way for its real-time deployment and low-power operation on edge devices, contributing to the intelligent transformation and sustainable development of agricultural production.

RLK-YOLOv8 provides a robust solution for automated strawberry detection and yield estimation, offering significant improvements over existing algorithms. Its integration into precision agriculture systems could help reduce labor costs, improve operational efficiency, and offer more accurate yield predictions. Future efforts will focus on enhancing real-time application performance, expanding its adaptability to varying environmental conditions, and exploring further deployment on edge devices to maximize its practical value in smart farming systems.

## Data Availability

The raw data supporting the conclusions of this article will be made available by the authors, without undue reservation.
